# Technology-based CBT in Reducing Symptoms of OCD in Children: A Systematic Review

**DOI:** 10.33790/jphip1100177

**Published:** 2021-05-12

**Authors:** Francine Samson, Barbara Tafuto, Nadina Jose, Lisa Palladino Kim

**Affiliations:** 1 Rutgers University, School of Health Professions, 65 Bergen St. Newark NJ, 07107.United States.

**Keywords:** Obsessive-Compulsive Disorder, Technology, Video-Conferencing, Internet, Cognitive Behavior Therapy, Child, Adolescents, COVID-19

## Abstract

**Background:**

Obsessive Compulsive Disorder (OCD) is an anxiety disorder affecting up to 3% of children and adolescent in the United States. Cognitive behavioral therapy (CBT) is the first-line treatment for mild to moderate OCD in children. Despite the benefits of CBT in treatment of OCD, only one-third of clinicians regularly use CBT compared to psychotropic medication due to lack of access. The COVID-19 pandemic has resulted in increased relapses in OCD severity and further limited access to traditional face-to-face CBT treatment due to health and safety precautions.

**Purpose:**

This systematic review aims to demonstrate the efficacy of technology-based CBT by evaluating results of clinical trials and its comparability to traditional CBT methods.

**Methods:**

An evidence-based search was conducted using terms such as “Obsessive-Compulsive disorder” AND “ internet” AND “cognitive behavior therapy” and “children”. A total of 716 articles were identified.

**Results:**

After screening titles, abstracts, and full articles for relevance, 7 studies with a total of 254 subjects and four different programs involving technology-based CBT were included in this systematic review. Information gathered in this review support the use of technology-based CBT as an effective treatment in reducing the severity of OCD symptoms as shown by statistically significant reductions in Children Yale-Brown Obsessive-Compulsive Scale scores. Additionally, the results from this review support previous clinical studies demonstrating that effects of technology-based CBT were non-inferior to traditional CBT methods.

**Conclusion:**

The results of this systematic review support the use of technology-based CBT for the treatment of OCD in children and adolescents, especially in times where access to in-person therapist sessions are not possible due to geographical or global concerns. Additional research is needed to understand the impact and acceptability of new CBT methods on daily-life as well as the effectiveness of technology-based methods on more severe cases of OCD.

## Introduction

Obsessive Compulsive Disorder (OCD) is an anxiety disorder affecting up to 3% of children and adolescents [1]. OCD is characterized by repetitive behaviors or compulsions, irrational thoughts, urges, and worries (obsessions), which can be severely disabling and anxiety-inducing for many patients [1]. Obsessions are defined as feelings of anxiety or other disturbing emotional experiences related to expectations of unfortunate outcomes. Compulsions are behaviors that are often linked to the individual’s obsessions in an attempt to neutralize the obsessive thoughts and prevent the feared outcome. OCD is associated with functional impairment and a lower quality of life. If left untreated, OCD can become a chronic illness with symptoms reoccurring overtime.

Treatment options for management of OCD in children and adolescents include psycho-education, relaxation training, counseling and/or behavioral interventions, family counseling, and medications [1]. Cognitive behavioral therapy (CBT) is the first-line treatment for mild to moderate OCD in children [2, 3]. Several randomized clinical trials (RCT) have shown that patients receiving CBT showed high rates of remission with large effect sizes. In addition, recent metanalysis support the use of CBT in combination with serotonin reuptake inhibitors (SRI) and is no better than CBT alone [2]. Evidence also supports long-term efficacy of CBT, lasting 9 months to 1 year, in the management of OCD in children and adolescents [2]. Despite the benefits of CBT in treatment of OCD, only one-third of clinicians regularly use CBT due to limited access, lack of highly trained therapists, geographical and financial barriers, and high costs [4].

With recent events stemming from the SARS-CoV-2 (COVID-19) pandemic, researchers have found a significant increase in the frequency of contamination obsessions, causing worsening or relapse of OCD symptoms [5]. However, health and safety measures implemented during the pandemic such as stay-at-home mandates and social distancing have further limited access to traditional face-to-face CBT sessions. In an attempt to increase access to psychotherapy, new options to deliver CBT treatment have been developed. Methods, such as telephone and internet-based CBT (TCBT and ICBT) have emerged in the last decade, providing increased access OCD treatment and additional resources. These methods have been explored in multiple studies reporting efficacy that is comparable to traditional in-clinic CBT modalities. Previous systematic reviews have provided evidence supporting the acceptability and effectiveness of ICBT for the treatment of depression and anxiety [6]. One previous systematic review investigating the efficacy of ICBT for pediatric OCD has shown favorable, but limited evidence [7]. The present systematic review aims to further explore the efficacy of technology-based CBT in reducing OCD severity in children and adolescents measured by changes in Children’s Yale-Brown Obsessive-Compulsive Scale scores by assessing current data and recent clinical studies.

## Method

### Search Strategy

An evidence-based search was conducted using the Preferred Reporting Items for Systematic Reviews and Meta-Analyses (PRISMA) methods. The databases searched were Cochrane, PubMed, and Elsevier and included studies published up to October 2020. The literature search included a combination of thesaurus and MeSH terms to identify references containing three main concepts: “Obsessive-Compulsive Disorder”, “internet or telephone cognitive behavior therapy”, and “children or adolescents”. Additional articles were identified using pearl growing strategies, such as checking reference lists of relevant articles, suggested similar articles, and related authors during the course of the review.

### Inclusion and exclusion criteria

Randomized controlled trials, open trials, pilot trials, non-blinded trials, and cohort studies with sample populations of 10 subjects or more published in English were included in the systematic review. Qualitative studies and reviews, commentaries, single-case studies, and studies having less than 10 subjects were excluded.

The PICO approach to literature reviews was used to identify inclusion and exclusion criteria on population, intervention, comparator, and outcomes. Trials that included children and adolescents up to 18 years of age with a primary diagnosis of OCD in accordance with standardized diagnostic criteria (DSM) were included. Clinical trials involving adults > 18 years of age or children with a secondary diagnosis of OCD were excluded. Trials assessing the use of technology-based CBT, such as those involving teleconferencing, videoconferencing, and/or internet-based modules were selected. Studies assessing technology-based CBT against traditional face-to-face CBT or no CBT were included in this systematic review. Studies assessing severity of OCD symptoms as measured by the Children’s Yale-Brown Obsessive-Compulsive Scale (CY-BOCS) were included in the review.

### Final Search Syntax

The final search syntax used to conduct the current systematic review was as follows:

1. (Obsessive-Compulsive Disorder) OR (OCD) OR (Anankastic Personality) OR (Neurosis, Obsessive-Compulsive)
2. (Internet) OR (Telephone) OR (Web) OR (Video) OR (Technology)
3. 1 AND 2
4. (Behavior Therapy) OR (Behavior Modification) OR (Conditioning Therapy) OR (Therapy, Behavior) OR (Therapy, Conditioning)
5. 3 AND 4
6. (Children) OR (Pediatric) OR (Minor)
7. 5 AND 6
8. (Severity) OR (Children’s Yale-Brown Obsessive-Compulsive Scale)
9. 7 AND 9

## Results

### Search Results

**Figure 1.**
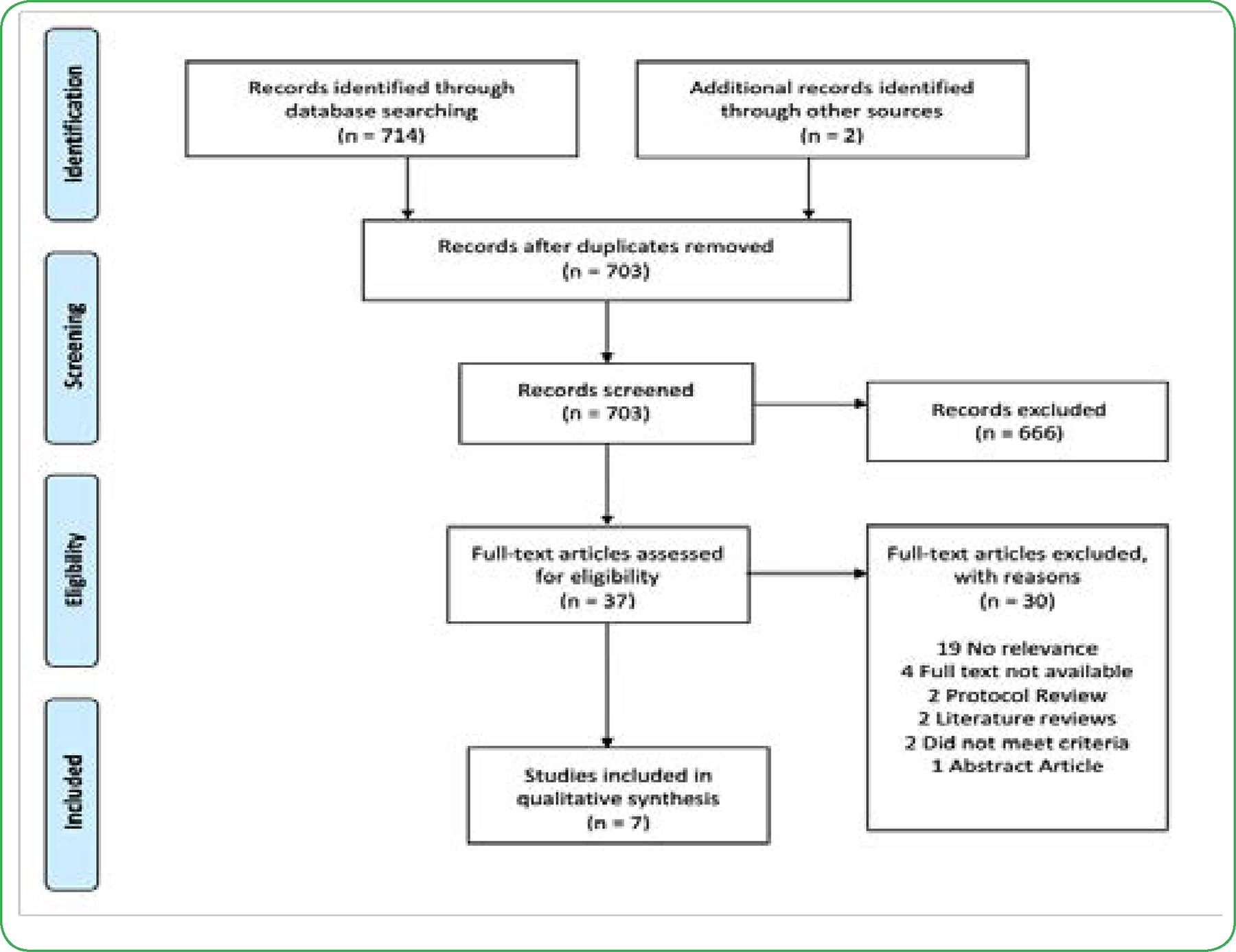
PRISMA Flow Diagram. Process of literature search (Flowchart taken from Moher)[8]

The initial database search identified 716 potential references. 13 duplicate articles were removed, resulting in 703 records to be screened. Of the remaining references, 666 were excluded after screening titles and abstracts, leaving 37 articles to be assessed for eligibility. 30 articles were further excluded due to access limitations or inability to meet inclusion criteria. Finally, seven (7) research studies were included in this systematic review (see Figure 1 for PRISMA Flow Diagram).

### Study Summary

Eligible studies included a total of 254 subjects, of which 56% were male on average (Table 1). The mean age ranged from 6.65 [9] to 14.97 [10] years old. The percentage of participants in each treatment arm who had other anxiety-related comorbid diagnoses ranged from 39% [10] to 100% [11]. In two studies, half or more of the participants also received psychotropic medication in addition to CBT for treatment of OCD symptoms [11, 12]. The mean CY-BOCS score across all studies was 23.85 and ranged from 21.3 [13] to 29.1[11].

Included studies were conducted in Sweden, United States, United Kingdom, and Australia [9–15] (Table 2). Of the included studies, three were randomized clinical trials (RCTs) [9, 10, 12, 15] two were open trials [14, 13] and one was a multiple baseline-controlled trial [11]. In the randomized trials, two studies investigated the efficacy of technology-based CBT versus traditional in-clinic sessions [9,15]. Additionally, studies by Lenhard and Storch compared the effects of technology-based CBT versus a waitlist control arm in which patients did not initially receive CBT therapy [10, 12].

There were several types of technology-based CBT utilized in the studies. In a study conducted by Turner, et al., treatment consisted of 14 CBT sessions conducted either in-person or via telephone [10]. In another randomized clinical trial, Storch utilized webcam-based CBT as a means to provide needed therapy. In this design, handouts were e-mailed to participants and family members before each scheduled session, homework assignments were discussed during the sessions or e-mailed to the therapist upon completion [15]. Comer, et al. implemented a similar approach, using an internet-based video-teleconferencing platform to allow therapists to remotely deliver real-time treatment. To replace the typical in-person activities performed in clinic-based CBT, interactive computer games were used to enhance understanding of treatment concepts [12]. After either a one-to-two week intensive treatment session, Farrell et al. implemented a 3-week e-therapy maintenance program in which therapists would video call participants once a week to follow-up on progress [9]. In three separate trials, Lehnard and Aspvall implemented an internet-based CBT platform named “BiP [BarnInternetProjektet] OCD designed for use by both adolescents and their parents [11]. This CBT program consisted of 12 chapters filled with animations and interactive modules with regular web-based contact with the therapist to guide children.

### Efficacy

**Figure 2.**
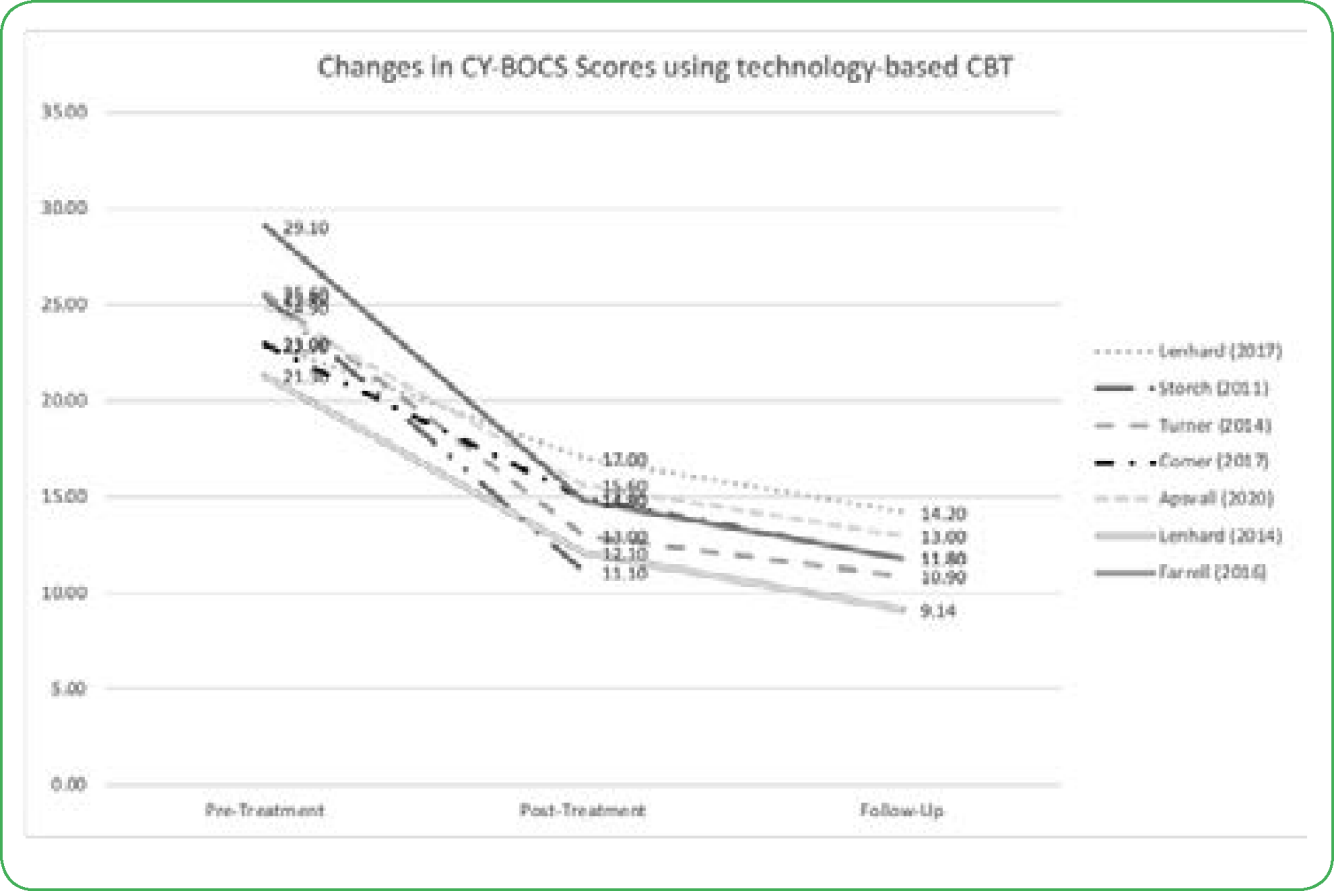
Changes in CY-BOCS Scores Using Technology-based CBT

**Figure 3.**
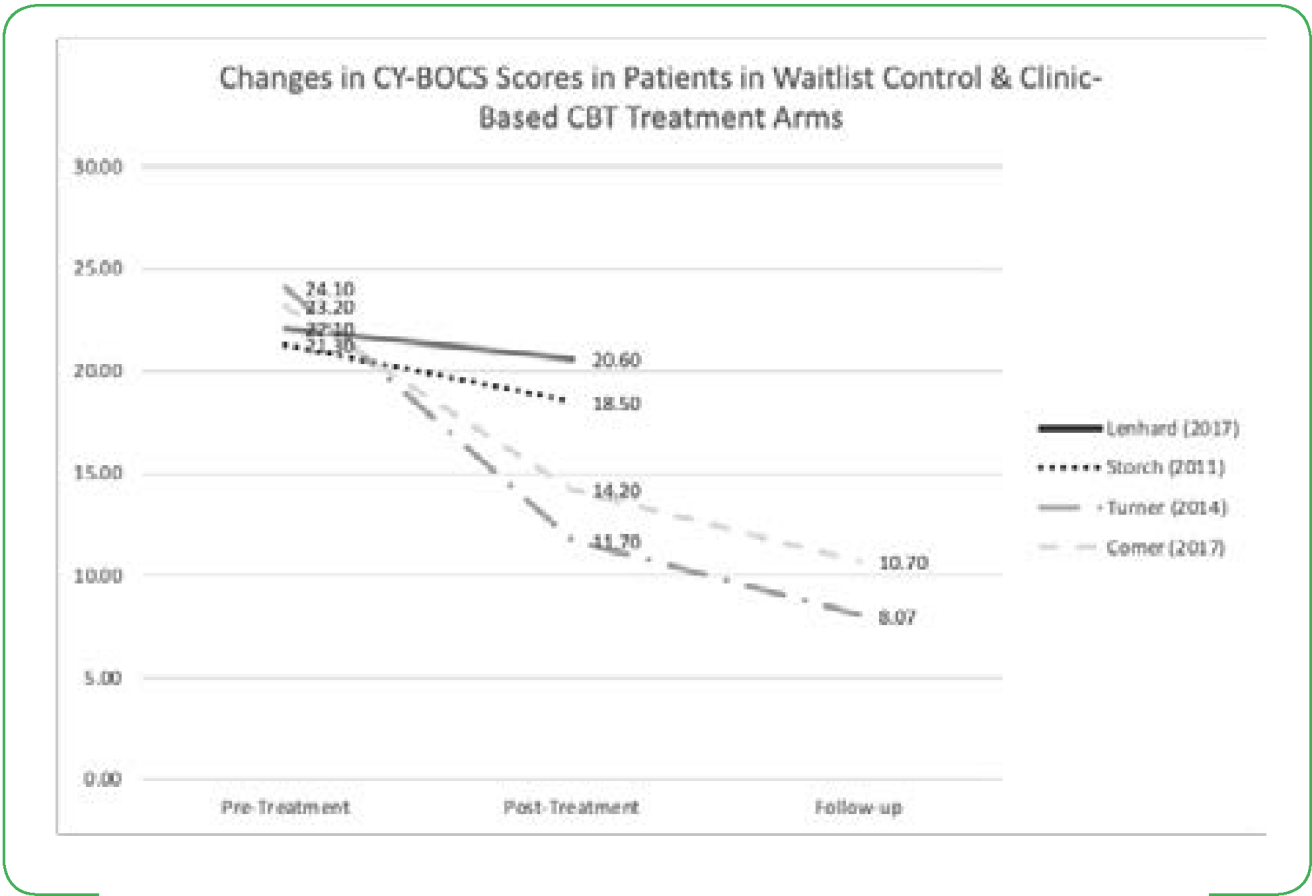
Changes in CY-BOCS Scores in Patients in Waitlist Control & Clinic-Based CBT Treatment Arms

All studies utilized the Children’s Yale-Brown Obsessive-Compulsive Scale to assess the severity of OCD symptoms from baseline/pre-treatment to post-treatment and follow-up. From pre-treatment to post-treatment, six studies reported a statistically significant decrease in CY-BOCS scores in within-group analyses (Figure 2) [9–14]. Storch, et al. [12] saw a significant CY-BOCS reduction in the webcam-based CBT (p<0.001) versus the waitlist-control group [12]. Additionally, Lenhard saw a similar effect where the internet-based CBT group had significantly lower severity scores compared with the waitlist control arm (Figure 2 and 3) [10]. These results indicate that technology-based CBT is superior to no-CBT approaches with large between-group effect sizes. Both Turner and Comer reported no significant differences in results from technology-based CBT versus traditional clinic-based CBT, indicating that technology-based CBT was non-inferior to CBT (Figure 2 and 3) [9,15]. Research also showed that decreases in CY-BOCS scores taken at post-treatment were also maintained during various follow-up timepoints (Figure 2) [10,11,13–15].

### Risk of Bias

The Cochrane Collaboration’s tool was used to assess any possible risk of bias among the included studies. All studies showed possible risk of bias (Table 4). A high risk of bias was associated with blinding of participants in all studies due to the fact that treatment allocation was apparent, despite randomization in some studies. Therefore, blinding of participants was not possible for CBT methods [9,10,12,15]. A low risk of bias was observed across all studies as independent blinded raters assessed the primary and secondary outcome measures.

## Discussion

Based on the current research, this is the second systematic review studying the efficacy of technology-based CBT on children with OCD. The previous review supported the use of internet based CBT in children [7]. The purpose of this systematic review was to further demonstrate the efficacy of technology-based CBT using recent data and its implementation during the coronavirus pandemic. Seven research studies were identified using PRISMA search methodologies involving four different approaches to technology-based CBT with a total 254 children and adolescents.

According to the CDC, SARS-CoV-2 (COVID-19) pandemic has become the greatest international health crisis in the modern era [16]. Recent data has demonstrated that there has been a worsening of symptoms and increased relapse rates in patients suffering from OCD [5]. Due to concerns regarding safety of patients and social distancing rules, discussion on how to safely provide CBT therapy during this time is needed. With advances in technology, internet or telephone-based CBT has become increasingly available in recent years [7]. Previous research in adult populations has supported the use of internet-based CBT in treating depression and anxiety [6]. However, there is limited data available for treatment of pediatric OCD. The results of the current review further support the use of technology-based CBT in reducing the severity of OCD symptoms in children and adolescents. All studies showed statistically significant reduction in OCD severity, based on CY-BOCS scores [9–15]. There was limited data available on the accommodation, acceptability, and feasibility of various CBT methods.

Generally, there were low drop-out rates of participants, suggesting good acceptability and accommodation of these methods. In randomized clinical trials, effects of technology-based CBT were noninferior to clinic-based CBT further providing support of technology-based CBT as an effective treatment method for OCD [9,15].

Despite the growing amount of evidence supporting the use of new CBT methods, there is limited data regarding long-term maintenance and remission rates on patients. Current evidence showed that CBT effects were maintained at various follow-up timepoints (3–6 months post-treatment); however, only one study assessed OCD severity at 12-months post-treatment [9–11, 13–15 ]. Further research is needed to determine the long-term efficacy and sustainability of technology-based CBT.

A major limitation of current research is the low number of available studies and the small sample sizes. All eligible studies were performed in a select number of countries including, the United States, Sweden, United Kingdom, and Australia. Thus, there was a low rate of diversity in the patient demographics, jeopardizing the external validity and generalizability of the data. In addition, most patients who participated in the studies were diagnosed with mildto-moderate OCD at baseline. Data regarding use of these methods in patients with severe OCD is limited at this time. However, the fact that patients with other comorbidities were able to participate in the studies increased the external validity of results as this provides realistic manifestations of OCD in patients with multiple disorders as well as those being treated with other psychotropic medications.

The median age among the 7 studies ranged from 6.65–14.97 years; however, results of the studies did not account for differences between age groups. One study evaluated VT-CBT in treating early childhood OCD and demonstrated clinically significant improvements in children 4 to 8 years old [17]. In another study, e-therapy maintenance was shown to be effective in improving OCD symptoms in adolescents between 11 to 16 years old [11]. The results of the trials suggest that technology-based CBT is effective in treating children and adolescents between 4 and 18 years of age; however, additional research may be needed to understand the effect of age on understanding and applying CBT concepts delivered via internet and teleconferencing and consider whether technology-based methods is comparably effective across various age groups.

Another concern with the current research is in regard to the various CBT methods implemented. Technology-based CBT varied from simple telephone calls with the therapist to weekly interactive internet modules. There is limited data to support the efficacy of one method over another. In addition, studies involving video teleconferencing or internet-based modules required access to high-speed internet as part of the inclusion criteria [10,12–14,17]. Only one study provided minimum computer requirements such as a hard drive, webcam, and microphone, to standardize the delivery and experience of video teleconferencing [17]. Variations in technology may impact the quality and delivery of the treatment program and minimum technological requirements should be identified in future trials. Each method of technology-based CBT varied in the amount of time participants spent in direct contact with their assigned therapist as well as varied in parental involvement with the program. Concerns regarding patient privacy and confidentiality must also be taken into account to ensure the safety and security of patient-related health information. Based on current studies, this has become an unclear risk and should be addressed in future research. Such details need to be taken into consideration to determine the effectiveness and acceptability of the program.

A recent report from the Condition of Education confirms that 94% of 3-to-18 year olds have access to the internet at home [18]. Thus, technology-based CBT provides a potential solution for increasing availability to therapy for patients who do not currently have access to in-clinic treatment due to geographical, socioeconomical or pandemic-induced concerns. These methods may also result in better adherence to treatment programs and lower drop-out rates due to ease of convenience. Additionally, the use of modern technology, such as smartphones, computers, and interactive applications may result in increased understanding of concepts related to CBT and increase cost-effectiveness of CBT therapy as it may be easier to incorporate these methods into everyday life. More research is required to further understand the geographical, financial, and long-term impacts of new technology-based CBT as well as the efficacy of these programs in patients with more severe symptoms of OCD.

## Conclusion

The results of this systematic review indicate that technology-based CBT is effective in reducing severity of OCD symptoms as measured by CY-BOCS scores. Such methods using technology-based CBT are non-inferior to traditional CBT and superior to no-CBT treatment. The results also support the use of technology-based CBT in helping children and adolescents overcome relapses in OCD-symptoms due to the recent COVID-19 pandemic. Although limited data exists on the feasibility and acceptability of technology-based therapy, the results provide support for the idea that technology-based CBT can be implemented when access to in-clinic therapy is not possible due to heightened obsessions or fears or social-distancing mandates. Larger, multi-national studies are required to understand the ability of technology-based methods on overcoming geographical and logistical barriers of CBT as well as long-term maintenance of e-therapy.

## Figures and Tables

**Table 1:** Patient characteristics.

	Lenhard(2017)		Storch(2011)		Turner(2014)		Comer(2017)		Aspvall(2020)	Lenhard(2014)	Farrell(2016)
	Waitlist Control	ICBT	Waitlt Contol	W-CBT	CBT	TCBT	CBT	VTCFB-CBT	ICBT	ICBT	CBT with e-therapy maintenace
Toal (N)	34	33	15	16	36	36	22		31	21	10
Median Age(Year)	14.97	14.21	11.2	11	14.5	14.19	6.65		14	14.4	13.6
Gender(% Male)	59%	48%	60%	63%	52.80%	55.60%	59.10%		67.70%	38.10%	60%
Presence of comorbid diagnosis (%)	47%	39%	Not Reported		Not Reported		81.20%		54.80%	71.40%	100%
Current Psychotrophic Medication(%)	21%	27%	53%	56%	22.60%	17.90%	Not Reported		22.60%	19.10%	50%
CY-BOCS Baseline Total Score, Mean (SD)	22.1(3.91)	23.0(4.31)	25.4(3.61)	21.3(2.74)	24.1(4.02)	25.6(3.86)	23.2(3.2)	22.9(4.1)	24.6(3.5)	21.3(3.54)	29.1(4.18)

Abbreviations ICBT, Internet Congnitive Behaviour Therapy; SD,Standard Deviation; VTC-FB-CBT,Video Teleconferencing Family Based-Congnitive Behaviour Therpy; W-CBT,Webcam-Congnitive Behaviour Therpy

**Table 2.** Study Summary Table

Author Year	Country	Purpose	Study Design	Intervation	Control	Efficacy Outcome Measures	Acceptabliity/Feasibility Outcome Measures	Outcome Timepoints
Lenhard(2017)	Sweden	Treatment efficacy	Randomized Controlled trial	Internet-based CBT	Waitlist Control	Cy-BOCS	Not applicable	Pretreatment, Posttreatment,3-Month Follow-up
						CGI-S		
						ChOCI-R		
						EWSAS		
						SCAS-S-C/P		
						CDI-S		
						FAS-PR		
Storch (2011)	United States	Treatment Efficacy	Randomized Controlled trial	Webcam-CBT	Waitlist Control	CY-BOCS	Not applicable	Baseline, Post-Treatment
						CGI-Severity		
						CGI - Improvement		
						COIS-C/P		
						MASC		
						FAS		
						CDI		
Turner(2014)	United Kingdom	Treatment efficacy acceptability	Randomized Controlled non-Inferiority trial	Telephone CBT	Clinic-based CBT	CY-BOCS	Treatment Satisfaction	Baseline, , Post-Treatment, 3-Month Follow-Up, 6-Month Follow-Up, 12-Month Follow-Up
						ChOCI-R	Treatment credibility and expectancy scale	
						BDI-Y		
						SDQ		
						DASS	Treatment Satisfaction	
						CGAS		
						CGI-I		
						FAS		
Comer (2017)	United Kingdom	Treatment Efficacy Acceptability Feasibility	Pilot randomized trial	Video teleconferencing-family-based-CBT	Clinic-based CBT	CY-BOCS	WAI	Pre-Treatment, Week 1,Intensive Tx Week 2, E-therapy Week 1, E-therapy Week 2, E-therapy Week 3, 1-month Follow-Up, 6-month Follow-Up
						CGI-S/I	Therapist Feedback	
						CGAS		
						FAS-PR		
Aspvall (2020)	United Kingdom, Sweden, Australia	Treatment acceptability feasibility, and efficacy	Open trial	Internet-based CBT	N/A	CY-BOCS	Therapist Feedback	Baseline, Post-Treatment,3-Month Follow-Up
						CGAS		
						ChCOI-R-P		
						WSPAS-Y/P		
Lenhard (2014)	Sweden	Treatment acceptability feasibility, and efficacy	Open trial	Internet-based CBT	N/A	CY-BOCS	Patient acceptability survey	Pre-Treatment, PostTreatment, 3-Month Follow-Up, 6-Month Follow-Up
						ChCOI-R		
						COIS-R		
						CGI-I		
						CGAS		
						SCAS C/P		
						CDI-S		
						SDQ		
						FAS-PR		
Farrell(2016)	Australia	Treatment efficacy	Multiple baseline controlled trial	Intensive face-to-face CBT followed by e-therapy maintanence	N/A	CSR	Not applicable	Pre-Treatment, Post-Treatment, 6-Month Follow-Up
						CY-BOCS Total		
						CY-BOCS P		
						CGI-S		
						CDI		
						MASC		
						PedsQL		

**Table 3.** Study Outcomes Table

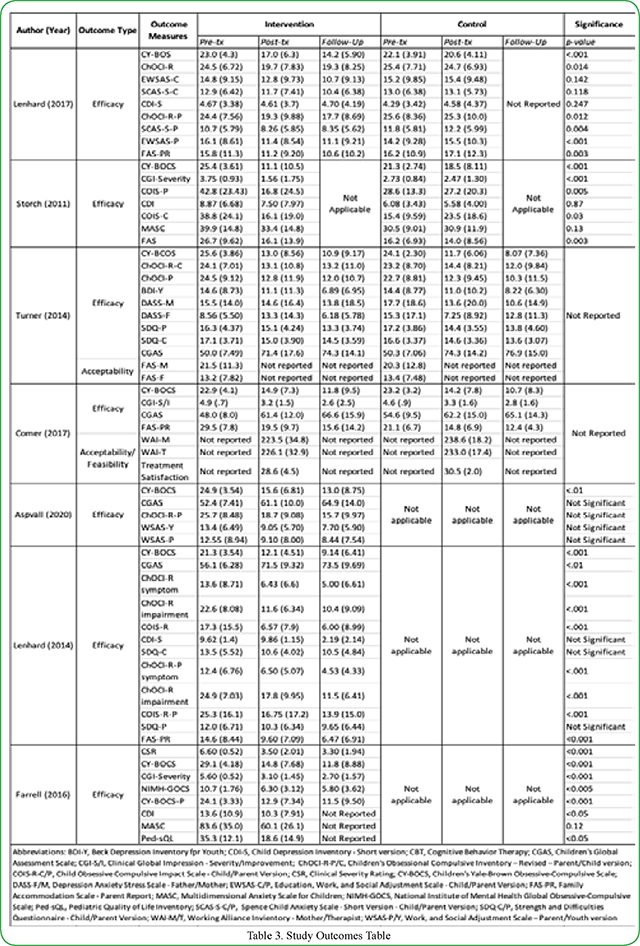

**Table 4.** Risk of Bias Table

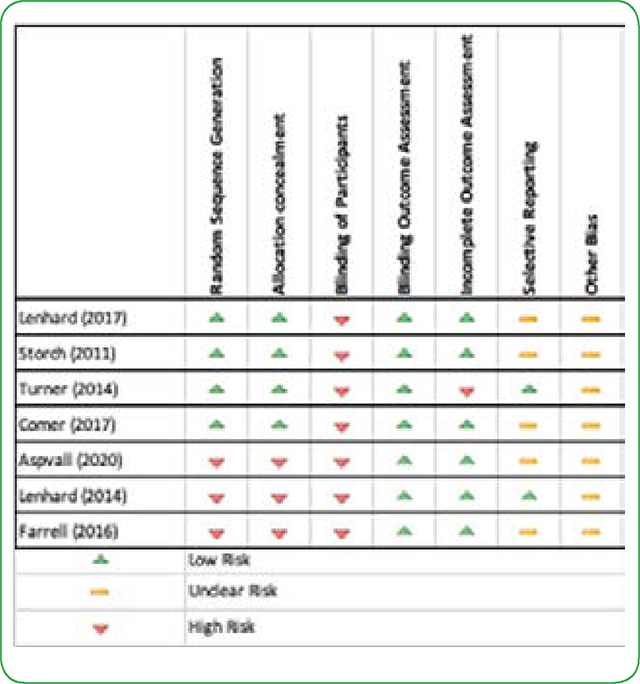
